# Panel containing three serum microRNAs: a promising biomarker for early detection of bladder cancer

**DOI:** 10.3389/fonc.2024.1470457

**Published:** 2024-12-24

**Authors:** Zhenjian Ge, Shengjie Lin, Chong Lu, Yong Xia, Rongkang Li, Xinji Li, Chen Sun, Zhenyu Wen, Wenkang Chen, Yingqi Li, Mingyang Li, Yu Lin, Jing Dong, Lingzhi Tao, Ling Ji, Yongqing Lai

**Affiliations:** ^1^ Department of Urology, Peking University Shenzhen Hospital, Shenzhen, China; ^2^ Institute of Urology, Shenzhen Peking University-The Hong Kong University of Science and Technology Medical Center, Shenzhen, China; ^3^ Shenzhen Clinical Research Center for Urology and Nephrology, Shenzhen, China; ^4^ Shantou University Medical College, Shantou, China; ^5^ The Fifth Clinical Medical College of Anhui Medical University, Hefei, China; ^6^ Department of Laboratory Medicine, Peking University Shenzhen Hospital, Shenzhen, China; ^7^ Shenzhen University Health Science Center, Shenzhen, China

**Keywords:** microRNA, biomarker, bladder cancer, MiR-212-3p, miR-30c-5p, miR-206

## Abstract

**Background:**

Bladder cancer (BC) is a common tumor worldwide. Screening for BC currently lacks a highly efficient, non-invasive, and inexpensive method. Serum microRNA (miRNA), which is stable and commonly present, has the potential to serve as a novel marker for BC diagnosis.

**Materials & methods:**

Based on a study involving 112 BC patients and 112 healthy subjects, we conducted this research in three phases to identify applicable microRNAs (miRNAs) in serum for BC diagnosis using quantitative reverse transcription polymerase chain reaction (qRT-PCR). A panel with optimal diagnostic value was developed. Additionally, we used bioinformatic analysis to explore the potential biological functions of the crucial miRNAs.

**Results:**

The diagnostic panel consisted of miR-212-3p, miR-30c-5p, and miR-206, with an area under the curve (AUC) of 0.838, sensitivity of 83.33%, and specificity of 73.81%. Furthermore, ATF3, GJA1, JPH2, MVB12B, RUNX1T1, SLC8A1, SPATA6, and TPM3 may be potential target genes of these three miRNAs.

**Conclusion:**

We developed a three-miRNA panel that could serve as a highly efficient and inexpensive biomarker for BC diagnosis and screening.

## 1 Introduction

Bladder cancer (BC) is one of the top 10 cancer types in terms of estimated cases and deaths for both men and women worldwide, responsible for an estimated 573,278 new cases and 212,536 deaths in 2020 (1). In developed regions and areas with a high prevalence of tobacco smoking, the incidence of BC is increased (2). Non-muscle invasive bladder cancer (NMIBC) accounts for more than 70% of BC cases, with a recurrence rate of about 50%-70%, and may progress to muscle-invasive bladder cancer (MIBC) in about 10%-20% of cases (3). The preferred treatment for BC is the removal of the tumor, typically done by inserting a transurethral device into the bladder (transurethral resection of the bladder tumor). Based on the depth of tumor invasion into the bladder wall and the tumor’s aggressiveness, chemotherapy, radiotherapy, or immunotherapy can be selected singly or in combination (4). Most BC cases are NMIBC, where tumors have breached the epithelial basement membrane of the bladder, and these patients have a 5-year survival rate of up to 90% (5). NMIBCs are prone to recurrence (50%-70%) but progress slowly. However, MIBC patients have a worse overall survival rate, with less than 50% surviving at five years. Distant metastases are more likely when BC cells breach the muscle layer, underscoring the importance of early diagnosis in improving survival rates (6). Therefore, early screening and diagnosis of BC before metastasis is critical to enhancing patient survival. One significant problem related to the diagnosis of BC is that standard diagnostic approaches, such as ultrasound, cystoscopy, and urinary cytology, are unsatisfactory due to poor precision or high invasiveness (7). For instance, urinary cytology, which is minimally invasive, has a specificity of 90%-100% but a sensitivity of 40%-60%, whereas cystoscopy, despite being more accurate, carries a 10% risk of complications like urinary tract infection even when using a flexible cystoscope (8). Thus, developing new diagnostic tools with higher specificity and sensitivity for early screening and detection of BC is of great significance.

MicroRNAs (miRNAs), which inhibit or degrade mRNA translation, are a type of endogenous noncoding small RNA that bind to the 3’ untranslated regions of related mRNAs to regulate gene expression. Previous studies have discovered that miRNAs participate in the occurrence and development of various malignant tumors and could serve as diagnostic biomarkers. In recent years, many studies have shown that highly stable circulating miRNAs primarily exist in lipid or lipoprotein complexes, such as exosomes, microvesicles, or apoptotic bodies (9, 10). Additionally, acquiring serum is relatively easy, and detecting RNA can be readily accomplished using quantitative reverse transcription-polymerase chain reaction (qRT-PCR). Therefore, some specific serum miRNAs show promise as potential noninvasive diagnostic biomarkers for BC.

In our study, based on qRT-PCR, we evaluated potential serum biomarkers by examining microRNA expression profiles in BC patients. The purpose of our research was to identify crucial miRNAs and develop a microRNA panel with excellent diagnostic value in three phases. Firstly, in the training phase, 10 serum miRNAs were screened using BC pools and healthy control (HC) pools. Secondly, abnormally expressed miRNAs identified in the training phase were further validated in the validation phase. Finally, the miRNAs with validated expression differences were analyzed using backward stepwise logistic regression to develop an efficient panel for BC diagnosis. Additionally, we used bioinformatic analysis to investigate the potential biological functions of the crucial miRNAs.

## 2 Material and methods

### 2.1 Ethical statement and specimen selection

The Ethics Committee of Peking University Shenzhen Hospital reviewed and approved this study. The collection process of serum samples from subjects was conducted in accordance with the relevant regulations established by the committee. From November 2017 to June 2021, 224 subjects were recruited from Peking University Shenzhen Hospital, comprising 112 BC patients and 112 HCs. All BC patients selected for this study were histopathologically confirmed and had not received any treatment before specimen collection. The tumors of these BC patients were primary tumors, not metastatic tumors. Samples from the HCs were obtained from healthy volunteers, none of whom had a history of cancer or other acute or chronic diseases. None of the participants in this research had received any therapy before the collection of serum specimens. Each participant was informed about the study, expressed full understanding, and signed the informed consent form.

### 2.2 Research design

**Figure 1:**
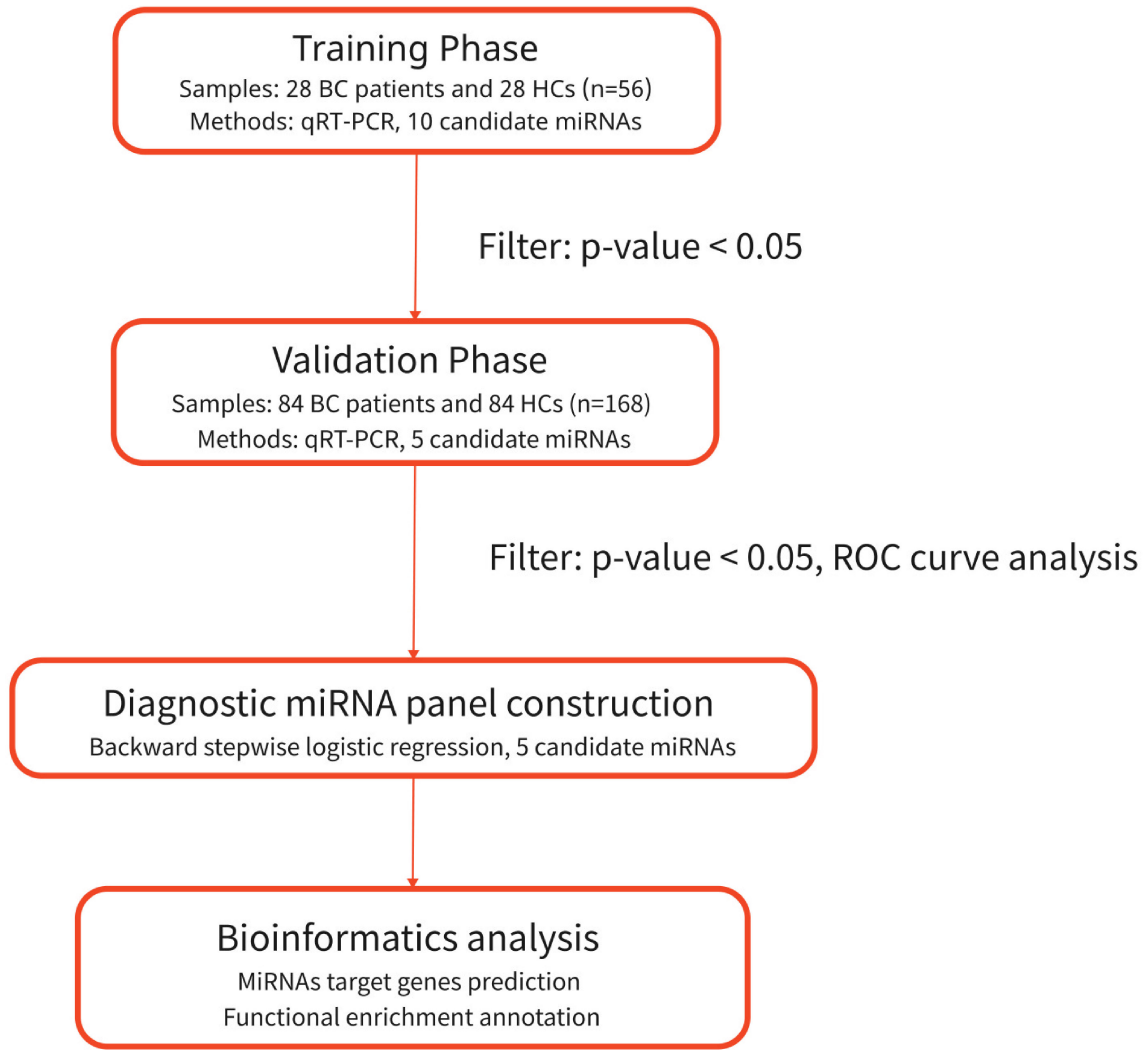
Overview of study design. BC, bladder cancer; HCs, healthy controls.

As shown in **Figure 1**, we designed a three-phase study. Initially, we selected candidate miRNAs with differential expression from GSE40355, published on Gene Expression Omnibus, as potential biomarkers. Then, we identified 10 miRNAs related to BC or other tumors that were differentially expressed in BC and normal control groups by searching the literature in PubMed. Next, we conducted training and validation phases to identify these candidate markers. In the training phase, we determined the expression of 10 candidate miRNAs in serum specimens from BC patients and HCs and selected differentially expressed miRNAs to proceed to the next stage. In the validation phase, we expanded the sample size and continued to perform qRT-PCR to confirm the expression of the miRNAs identified in the training phase. The samples consisted of 28 BC patients and 28 HCs in the training phase, and 84 BC patients and 84 HCs in the validation phase. Finally, we developed a miRNA-based panel with optimal diagnostic capability for BC using reverse stepwise logistic regression analysis.

### 2.3 RNA extraction

To normalize the variability in the assay process, 2 µL of miR-54 (cel-miR-54-5p, 10 nm/L, RiboBio) was added to each serum specimen at the beginning. Total serum RNA was then extracted using a TRIzol LS isolation kit (Thermo Fisher Scientific), followed by RNA concentration and purity determination using a NanoDrop 2000c spectrophotometer (Thermo Fisher Scientific).

### 2.4 RT-qPCR

Firstly, we used reverse transcription-specific primers from the Bulge-Loop miRNA RT-qPCR primer set (RiboBio) to amplify miRNAs and detect their expression profiles. Next, RT-qPCR was performed using the TaqMan probe on the LightCycler 480 Real-Time PCR system (Roche Diagnostics). The qPCR reactions were carried out at 95°C for 20 seconds, followed by 40 cycles of 95°C for 10 seconds, 60°C for 20 seconds, and 70°C for 10 seconds. Finally, to analyze the relative expression of the target miRNAs, we used the 2^−ΔΔCq^ method to calculate and plot the results (11).

### 2.5 Statistical analysis

For data analysis, we used SPSS software (SPSS 20.0 Inc), GraphPad Prism 8 (GraphPad Software Inc), and MedCalc (Version 19). Different optimal methods were utilized for data analysis according to different numerical traits. The Student’s t-test was employed to analyze continuous variables, and the χ2 test was used to analyze categorical variables. Additionally, multiple comparisons of data between different independent phases were implemented using the Kruskal-Wallis test or Mann-Whitney test. Statistical significance was defined as a p-value of less than 0.05. The diagnostic capability of the miRNAs was evaluated using the receiver operating characteristic (ROC) curve and the area under the ROC curve (AUC). The highest specificity and sensitivity were determined by the Youden index. Age at diagnosis was expressed as means ± standard deviations, and gender was expressed as numbers (percentages).

### 2.6 Bioinformatics analysis

To investigate the function of critical miRNAs in the development of BC, we utilized miRWalk2.0 (http://zmf.umm.uniheidelberg.de/mirwalk2) to predict a series of candidate miRNA targets. Additionally, miRWalk was used to predict and verify the interactions between miRNAs and their targets (12). In addition, we also use the Enrichr database (http://amp.pharm.mssm.edu/Enrichr/) to conduct enrichment analysis of target genes and functional annotation (13). Gene Ontology (GO) annotation and Kyoto Encyclopedia of Genes and Genomes (KEGG) pathways were also employed for target gene analysis in this study.

## 3 Results

### 3.1 Characteristics of participants

Overall, a total of 112 BC patients and 112 healthy subjects were included in this study. All BC patients were diagnosed using the TNM staging system and histologic classification based on the World Health Organization criteria. They were confirmed to have primary BC without metastatic tumors. None of the HCs had a history of cancer or other acute or chronic diseases. In both the training and validation stages, there were no statistically significant differences in age and gender between BC patients and HCs (**Table 1**). The parameters were expressed as numbers and percentages. Statistical comparisons were performed using the Wilcoxon-Mann Whitney test.

**Table 1:** Demographic manifestation of 224 participants (BC patients and HCs).

	Testing Phase (n=56)			Validation Phase (n=168)		
	BC	HCs		BC	HCs	
**Total Number**	28	28		84	84	
**Age at diagnosis**	65.5 ± 12.1	63.7 ± 9.1	p=0.586	58.3 ± 14.1	60.3 ± 15.1	p=0.394
**Gender**			p=0.528			p=0.373
Male	23 (82.14%)	20 (71.43%)		66 (78.57%)	60 (71.43%)	
Female	5 (17.86%)	8 (28.57%)		18 (21.43%)	24 (28.57%)	

Among two stages, there was no significant difference between BC and HCs in age and gender. Parameters were shown as number(percentage). Statistical contrast was exerted through the Wilcoxon-Mann Whitney test.

### 3.2 Candidate miRNAs screening

Based on the criteria of the adjusted p-value < 0.05 and |logFC| > 1, we identified abnormally expressed miRNAs using GEO2R (http://www.ncbi.nlm.nih.gov/geo/geo2r). Using the GSE40355 database, a total of 176 abnormally expressed miRNAs were screened out from 16 BC samples and 8 control samples, of which 92 were up-regulated and 84 were down-regulated. Furthermore, based on a literature search in PubMed, we selected 10 miRNAs relevant to BC or other cancers as candidate miRNAs for further investigation.

### 3.3 Differentially expressed miRNAs during the training phase

**Figure 2:**
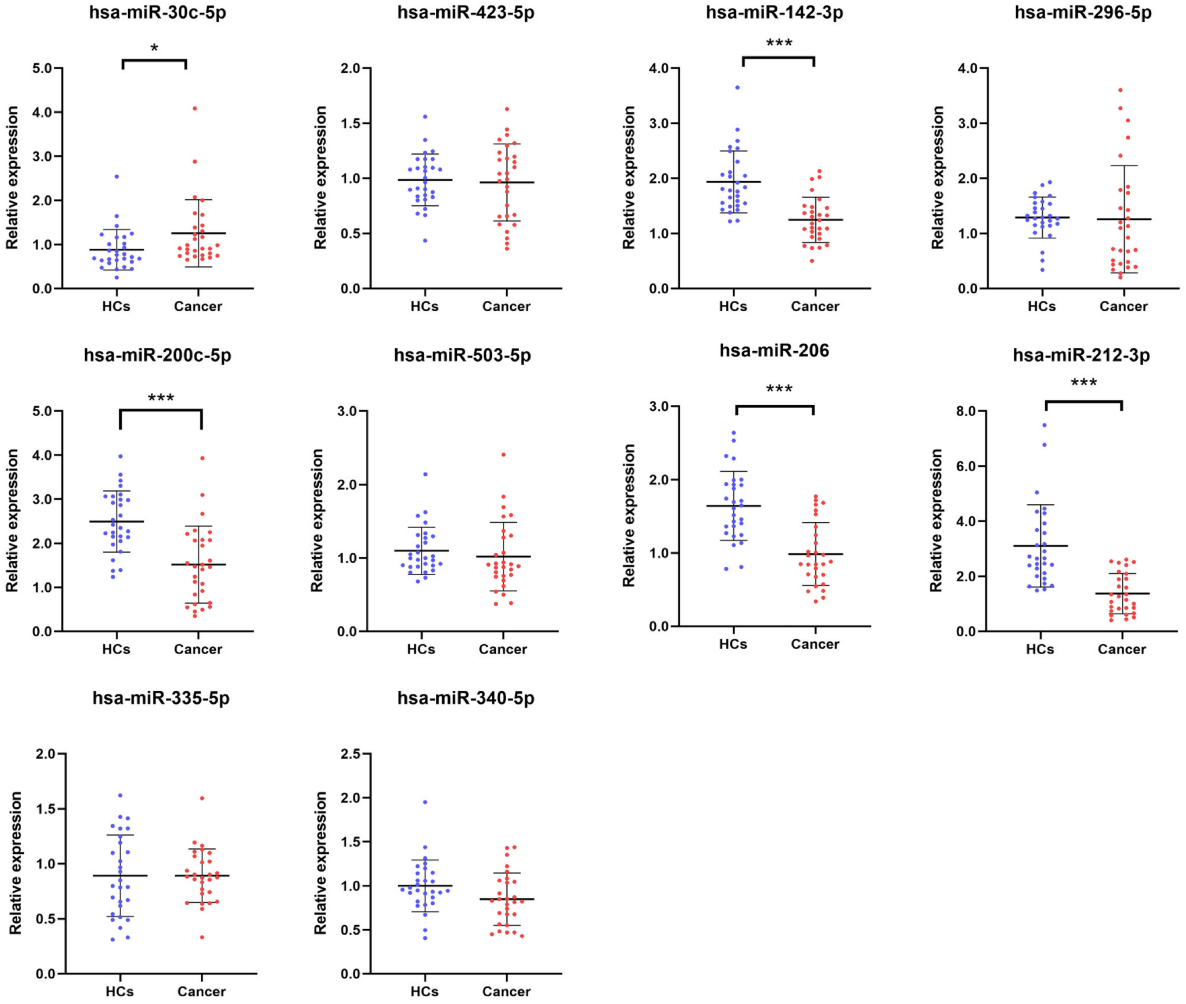
Relative serum expression of ten candidate miRNAs in training phase. Expression profiles of ten candidate miRNAs in serum samples from 28 BC patients and 28 HCs. Screening criteria: p-value < 0.05, *p < 0.05, ***p < 0.001. BC, bladder cancer; HCs, healthy controls.

Ten candidate miRNAs were selected and validated through RT-qPCR analysis in the training phase using samples from 28 BC patients and 28 HCs. The results are shown in **Figure 2**. Among these 10 miRNAs, 5 (miR-30c-5p, miR-142-3p, miR-200c-5p, miR-206, miR-212-3p) exhibited significant dysregulation between BC patients and HCs. Consequently, these 5 miRNAs were chosen for further investigation in the subsequent study.

### 3.4 Diagnostic capabilities of 5 miRNAs in the validation phase

**Figure 3:**
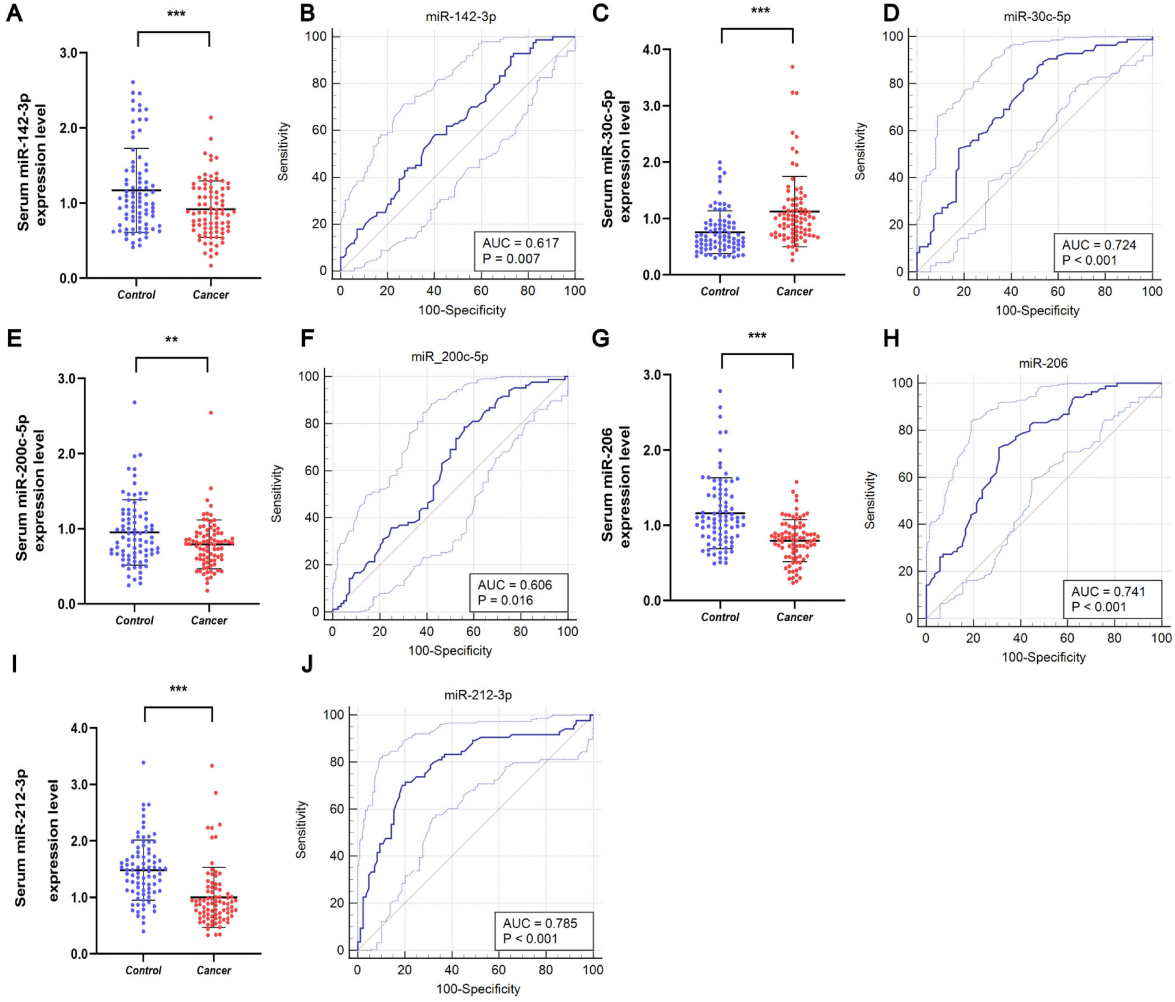
Serum expression and ROC curve analysis of five miRNAs in validation stage. Downregulated expression levels of **(A)** miR-142-3p, **(E)** miR-200-5p, **(G)** miR-206, and **(I)** miR-212-3p in the serum of BC patients compared to HCs, and **(C)** upregulated expression level of miR-30c-5p in the serum of BC patients. ROC curve analyses of **(B)** miR-142-3p, **(D)** miR-30c-5p, **(F)** miR-200-5p, **(H)** miR-206, and **(J)** miR-212-3p. **p < 0.01, ***p < 0.001.

These five miRNAs were further investigated using samples from 84 BC patients and 84 healthy subjects to assess their potential as serum markers for early BC screening. As shown in **Figure 3**, downregulation of miR-142-3p, miR-200c-5p, miR-206, and miR-212-3p was observed in the serum of BC patients, while upregulation of miR-30c-5p was observed in the serum of BC patients. To analyze the diagnostic capabilities of these five miRNAs, we plotted ROC curves.

The AUCs for the five miRNAs were determined as follows: 0.617 (95% CI: 0.539 - 0.691) for miR-142-3p, 0.724 (95% CI: 0.649 - 0.790) for miR-30c-5p, 0.606 (95% CI: 0.528 - 0.680) for miR-200c-5p, 0.741 (95% CI: 0.668 - 0.806) for miR-206, and 0.785 (95% CI: 0.715 - 0.845) for miR-212-3p (**Figures 3B, D, F, H, J**, respectively). Using the Youden index, we calculated the best cut-off values and listed the optimal specificity and sensitivity of these miRNAs for diagnosing BC in **Table 2**. The ROC curve analysis indicates that miR-212-3p, miR-30c-5p, and miR-206 exhibit strong diagnostic capabilities for BC, with AUC values of 0.785, 0.724, and 0.741, respectively.

**Table 2:** Outcomes of receiver operating characteristic curves and Youden index for 5 candidate miRNAs and the three-miRNA panel.

	AUC	P value	95% CI	Associated criterion	Sensitivity (%)	Specificity (%)
miR-142-3p	0.617	= 0.006	0.539 - 0.691	≤1.38	91.67	27.38
miR-212-3p	0.785	< 0.001	0.715- 0.845	≤1.01	70.24	80.95
miR-200c-5p	0.606	=0.015	0.528 - 0.680	≤0.95	78.57	44.05
miR-30c-5p	0.724	< 0.001	0.649 - 0.790	>0.62	89.29	46.43
miR-206	0.741	< 0.001	0.668 - 0.806	≤0.9	72.62	69.05
three-miRNA panel	0.838	< 0.001	0.774 - 0.890	>0.45422	83.33	73.81

AUC, area under curve; CI, confidence interval.

### 3.5 Development of the most suitable miRNA panel for BC

**Figure 4:**
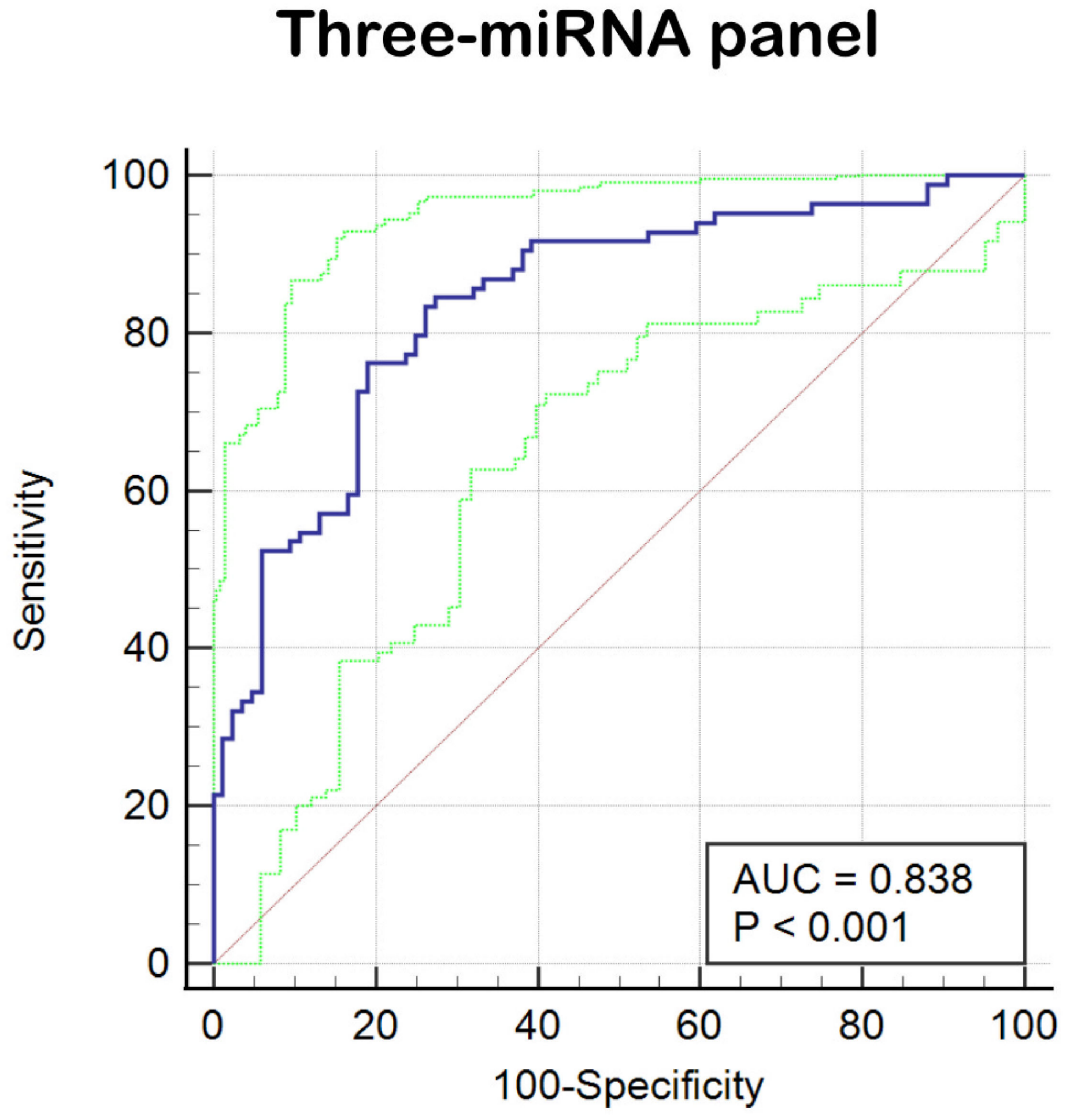
ROC curve analysis of three-miRNA panel (miR-212-3p, miR-30c-5p, and miR-206). The AUC of the panel is 0.838 (95% CI: 0.774–0.890; sensitivity = 83.33%, specificity = 73.81%).

In the final phase of our study, we verified that miR-212-3p, miR-30c-5p, and miR-206 possess superior diagnostic value for BC. Recognizing that combining multiple miRNAs can potentially enhance accuracy over single miRNA markers, we developed an optimized diagnostic panel. Integrating the expression profiles of these miRNAs from the last phase, we used a stepwise logistic regression model to establish the optimal panel for BC diagnosis. The formula of the model is as follows: Logit(P) = 2.36 + (1.336 × miR-30c-5p) + (-2.037 × miR-206) + (-1.35 × miR-212-3p). As depicted in **Figure 4**, ROC curves were generated for the three-miRNA diagnostic panels, revealing improved diagnostic efficacy with an AUC of 0.838 (95% CI: 0.774-0.890). This AUC value was higher than that achieved by each individual miRNA alone. Additionally, the diagnostic panel exhibited a sensitivity of 83.33% and specificity of 73.81%, as detailed in **Table 2**.

### 3.6 Bioinformatics analysis of miRNA panel

**Figure 5:**
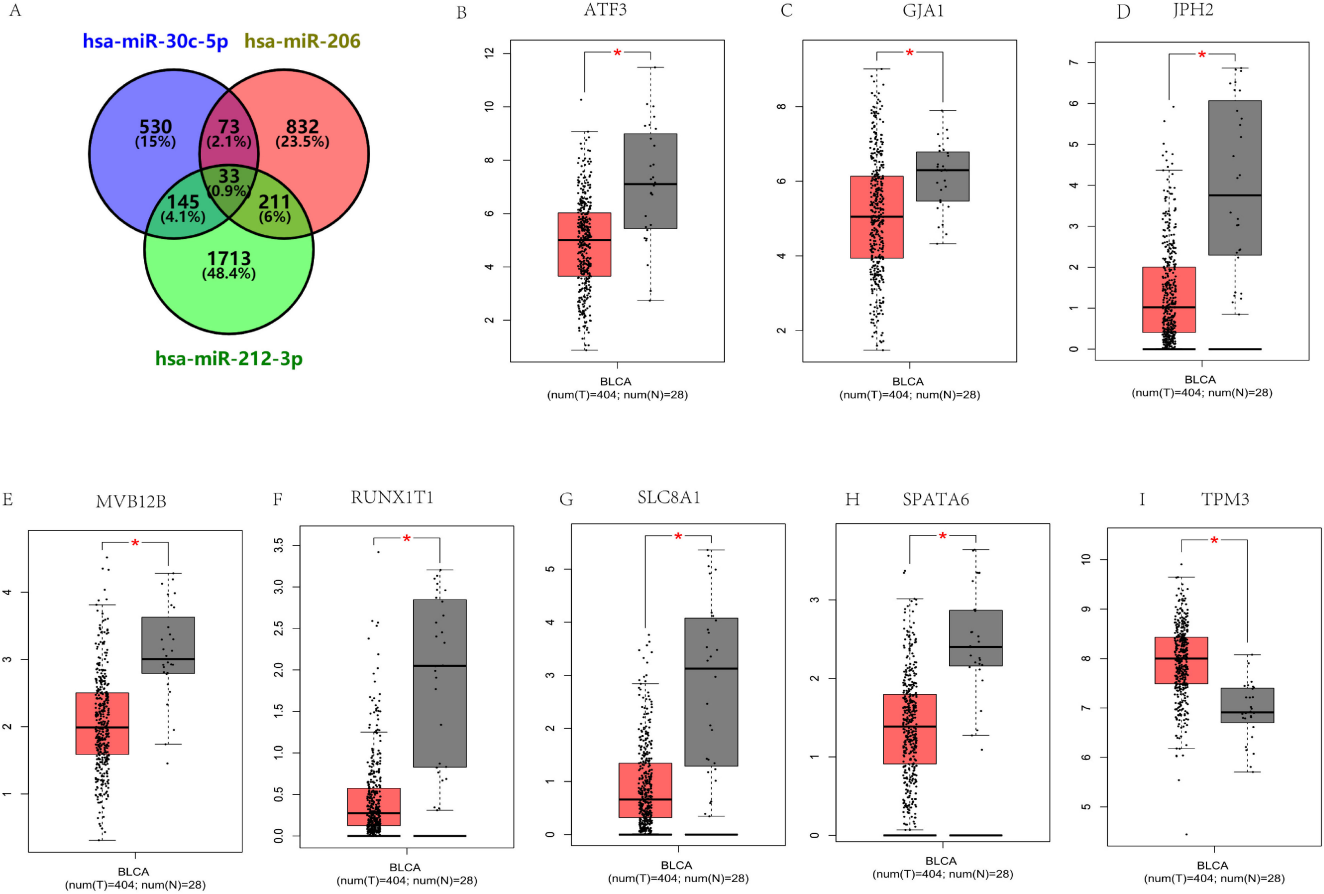
Prediction of target genes by miRWalk 2.0 for three critical miRNAs. **(A)** Venn diagram illustrating genes targeted by two or more miRNAs (a total of 462 genes). The expression of 33 putative genes targeted by all three miRNAs in 404 BC patients and 28 healthy controls was verified through GEPIA. Eight genes have abnormal expression in BC (|log2FC| > 1.5, p < 0.01): ATF3 **(B)**, GJA1 **(C)**, JPH2 **(D)**, MVB12B **(E)**, RUNX1T1 **(F)**, SLC8A1 **(G)**, SPATA6 **(H)**, TPM3 **(I)**. *p<0.01.

**Figure 6:**
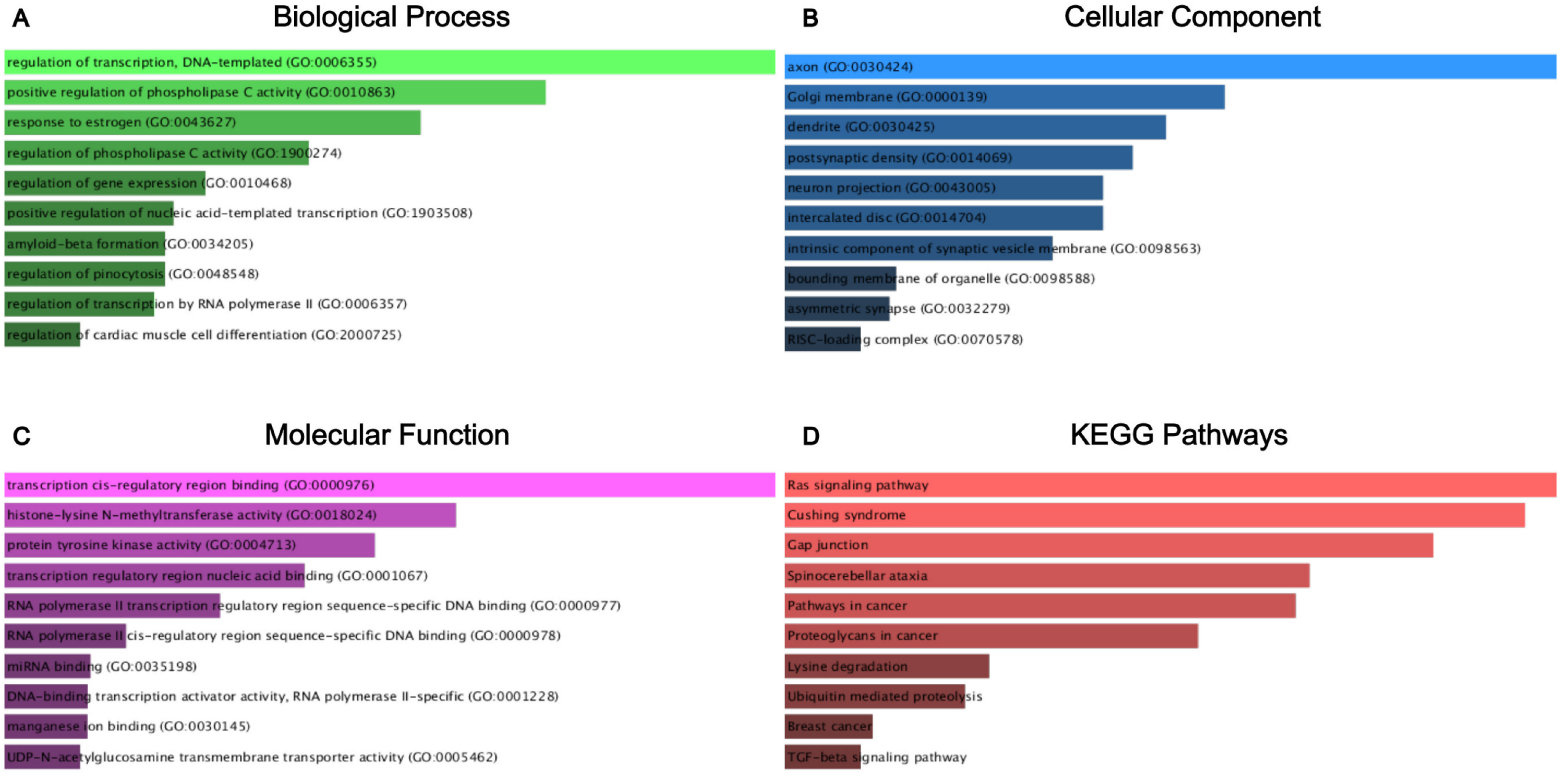
GO functional annotation and KEGG pathway enrichment analysis of the target genes of miR-212-3p, miR-30c-5p, and miR-206. **(A)** Biological process analysis; **(B)** cellular component analysis; **(C)** molecular function analysis; **(D)** KEGG pathway enrichment analysis.

After constructing the diagnostic panel using miR-212-3p, miR-30c-5p, and miR-206, we performed bioinformatics analysis to explore their potential target genes. Using MiRWalk2.0, we predicted potential target genes targeted by these miRNAs. Genes predicted by two or more miRNAs were selected, resulting in a total of 462 predicted genes. A Venn diagram illustrating the overlap of these genes is shown in **Figure 5A**. Eight genes, ATF3, GJA1, JPH2, MVB12B, RUNX1T1, SLC8A1, SPATA6, and TPM3, were identified as potential target genes for miR-212-3p, miR-30c-5p, and miR-206. Each of these genes exhibited a |log2FC| > 1.5 and p-value < 0.01 (**Figures 5B–I**). Subsequently, we conducted KEGG pathway enrichment analysis and GO annotation to further investigate these target genes. The top 10 enriched biological processes (BP), cellular components (CC), molecular functions (MF), and KEGG pathways are illustrated in **Figures 6A–D**. For instance, the analysis revealed significant enrichment in pathways such as the Ras signaling pathway, Cushing’s syndrome, gap junction, and spinocerebellar ataxia, highlighting their potential roles in BC pathogenesis.

## 4 Discussion

Studies have revealed that miRNAs play critical roles in cell metabolic pathways, intercellular communication, and modifying the tumor microenvironment. Using serum miRNAs for disease screening offers a non-invasive and convenient alternative to traditional inspection methods, making it potentially valuable in clinical practice (14, 15). Recently, several studies including Singh et al. (16), Qureshi et al. (17) and Dip et al. (18) revealed a potential relationship between miRNAs and bladder cancer, while their studies only showed the role of a specific miRNA in bladder cancer. Other studies including Li et al. (19) and Wen et al. (20) elucidated the potential of combined miRNA panel for diagnosis in bladder cancer or urothelial carcinoma. In our study, we designed a three-phase test—screening, training, and validation stages—to identify serum miRNAs that could serve as potential biomarkers for BC screening. We identified 5 miRNAs (miR-30c-5p, miR-142-3p, miR-200c-5p, miR-206, and miR-212-3p) with aberrant expression in serum between BC patients and HCs. Subsequently, we developed a three-miRNA-based signature comprising miR-212-3p, miR-30c-5p, and miR-206, which exhibited higher specificity and accuracy for BC screening compared to single miRNAs alone (AUC = 0.838; 95% confidence interval: 0.774 - 0.890). Additionally, we conducted bioinformatics analysis to explore the potential functions of these selected miRNAs.

In our diagnostic panel, miR-30c-5p was identified as overexpressed in the serum of BC patients, with an AUC of 0.724 (95% CI: 0.649-0.790). Recent studies have linked dysregulation of miR-30c-5p to advanced stage and overall survival outcomes in BC patients (21). This miRNA has been shown to be closely associated with the biological characteristics of tumor cells. For example, in glioma cells, miR-30c-5p could induce apoptosis and inhibit growth and invasion by targeting Bcl2, suggesting its potential as a therapeutic target to improve overall patient survival in glioma (22). Given these findings and our study, miR-30c-5p likely plays a significant role in the development and potential therapeutic targeting of BC as well.

Several studies have consistently demonstrated that miR-212-3p is downregulated in various types of tumors. It has been observed that miR-212-3p exerts anti-tumor effects by targeting NFIA, thereby inhibiting cell proliferation directly through NFIA regulation. It suggests that targeting NFIA with miR-212-3p could potentially serve as a therapeutic strategy for BC (23). Moreover, studies have indicated that overexpression of miR-212-3p can also suppress MAP3K3 protein expression, further inhibiting cell invasion, proliferation, and migration in certain cancers, such as high-grade serous ovarian cancer (HGSOC) (24). Combining these insights into its pathways and targets, the importance of miR-212-3p in BC screening was further validated, demonstrating an AUC of 0.785 (95% CI: 0.715-0.845).

Research has revealed that miR-206 plays a crucial role in regulating BC cell proliferation, invasion, and migration by targeting RMRP. The long non-coding RNA (lncRNA) RMRP was expressed at higher levels in BC cells compared to adjacent tissues and strongly associated with tumor size, lymphatic metastasis, and overall survival in BC patients (25). Furthermore, another study has demonstrated that miR-206 targets YRDC to suppress tumor development in BC (26). This dual role of miR-206 in regulating multiple pathways related to BC pathogenesis suggests its suitability as a serum marker for BC diagnosis, with an AUC of 0.741 (95% CI: 0.668–0.806).

After predicting target genes, a total of 8 genes met the criteria of |log2FC| > 1.5 and p < 0.01, including ATF3, GJA1, JPH2, MVB12B, RUNX1T1, SLC8A1, SPATA6, and TPM3 (**Figure 5**). Therefore, they might be potential target genes of the three miRNA-based panel. Studies have shown that ATF3 inhibits the metastasis of BC cells, and ATF3 up-regulates actin remodeling mediated by GSN. Thus, ATF3 and GSN could serve as prognostic markers for BC metastasis (27). Additionally, numerous studies have indicated that GJA1 could be a promising prognostic marker for poor survival and a therapeutic target in cervical cancer, suggesting a key regulatory role across various cancers (28). The positive feedback loop involving RUNX1T1 contributes to BC progression and may present a potential treatment strategy (29). CircSLC8A1 is implicated in the CircSLC8A1/miR-130b and miR-494/PTEN axes as a tumor suppressor, potentially offering therapeutic targets for BC treatment (30). Moreover, TPM3 is positively associated with Th2 cells and other immune cell infiltration in BC, influencing the development of BC through immune modulation (31). However, there has been limited research on the relationship between JPH2, MVB12B, SPATA6, and BC. In conclusion, these 8 genes play significant roles in the tumorigenesis and progression of BC and various other cancers. Further exploration into their specific roles in bladder carcinogenesis and progression is warranted.

Overall, our results were demonstrated to be credible and significant; however, several limitations should be addressed. Firstly, the sample size of both patient and control groups in this study was relatively small. Secondly, while there have been numerous BC-related miRNAs in serum, we only investigated 10 miRNAs in this study. Additionally, a more comprehensive bioinformatics analysis of the miRNAs in this experiment would enhance understanding about their clinical utility.

In summary, we developed a three miRNA-based panel (miR-212-3p, miR-30c-5p, and miR-206) with potential as novel and noninvasive serum markers for BC diagnosis and screening.

## Data Availability

The data presented in the study are deposited in the GEO repository, accession number GSE40355.

## References

[1] Sung H Ferlay J Siegel RL Laversanne M Soerjomataram I Jemal A. Global cancer statistics 2020: GLOBOCAN estimates of incidence and mortality worldwide for 36 cancers in 185 countries. CA Cancer J Clin. (2021) 71:209–49. doi: 10.3322/caac.21660 33538338

[2] Antoni S Ferlay J Soerjomataram I Znaor A Jemal A Bray F. Bladder cancer incidence and mortality: A global overview and recent trends. Eur Urol. (2017) 71:96–108. doi: 10.1016/j.eururo.2016.06.010 27370177

[3] Ahmadi H Duddalwar V Daneshmand S. Diagnosis and staging of bladder cancer. Hematol Oncol Clin North Am. (2021) 35:531–41. doi: 10.1016/j.hoc.2021.02.004 33958149

[4] Lenis AT Lec PM Chamie K. Bladder cancer. JAMA. (2020) 324:2006. doi: 10.1001/jama.2020.17601 33201205

[5] Prout GR Jr. Barton BA Griffin PP Friedell GH. Treated history of noninvasive grade 1 transitional cell carcinoma. The National Bladder Cancer Group. J Urol. (1992) 148:1413–9. doi: 10.1016/S0022-5347(17)36924-0 1433540

[6] Knowles MA Hurst CD. Molecular biology of bladder cancer: new insights into pathogenesis and clinical diversity. Nat Rev Cancer. (2015) 15:25–41. doi: 10.1038/nrc3817 25533674

[7] Usuba W Urabe F Yamamoto Y Matsuzaki J Sasaki H Ichikawa M. Circulating miRNA panels for specific and early detection in bladder cancer. Cancer Sci. (2019) 110:408–19. doi: 10.1111/cas.2019.110.issue-1 PMC6317958 30382619

[8] Almallah YZ Rennie CD Stone J Lancashire MJ. Urinary tract infection and patient satisfaction after flexible cystoscopy and urodynamic evaluation. Urology. (2000) 56:37–9. doi: 10.1016/S0090-4295(00)00555-0 10869618

[9] Kirschner MB Kao SC Edelman JJ Armstrong NJ Vallely MP van Zandwijk N. Haemolysis during sample preparation alters microRNA content of plasma. PloS One. (2011) 6:e24145. doi: 10.1371/journal.pone.0024145 21909417 PMC3164711

[10] Gopalan V Smith RA Lam AK. Downregulation of microRNA-498 in colorectal cancers and its cellular effects. Exp Cell Res. (2015) 330:423–8. doi: 10.1016/j.yexcr.2014.08.006 25128149

[11] Livak KJ Schmittgen TD. Analysis of relative gene expression data using real-time quantitative PCR and the 2(-Delta Delta C(T)) Method. Methods. (2001) 25:402–8. doi: 10.1006/meth.2001.1262 11846609

[12] Dweep H Gretz N. miRWalk2.0: a comprehensive atlas of microRNA-target interactions. Nat Methods. (2015) 12:697. doi: 10.1038/nmeth.3485 26226356

[13] Kuleshov MV Jones MR Rouillard AD Fernandez NF Duan Q Wang Z. Enrichr: a comprehensive gene set enrichment analysis web server 2016 update. Nucleic Acids Res. (2016) 44:W90–97. doi: 10.1093/nar/gkw377 PMC4987924 27141961

[14] Piperigkou Z Karamanos NK. Dynamic interplay between miRNAs and the extracellular matrix influences the tumor microenvironment. Trends Biochem Sci. (2019) 44:1076–88. doi: 10.1016/j.tibs.2019.06.007 31288968

[15] Zhang C Zhao Y Xu X Xu R Li H Teng X. Cancer diagnosis with DNA molecular computation. Nat Nanotechnol. (2020) 15:709–15. doi: 10.1038/s41565-020-0699-0 32451504

[16] Singh P Singh A Gupta N Raja KD Singh P Agarwal S. Non-invasive diagnostic potential of microRNA-203 in liquid biopsy of urothelial carcinoma of bladder. Mol Cell Biochem. (2022) 477:2173–82. doi: 10.1007/s11010-022-04431-2 PMC9021364 35445913

[17] Qureshi A Fahim A Kazi N Farsi Kazi SA Nadeem F. Expression of miR-100 as a novel ancillary non-invasive biomarker for early detection of bladder carcinoma. J Pak Med Assoc. (2018) 68:759–63. 29885177

[18] Dip N Reis ST Srougi M Dall’Oglio MF Leite KR. Expression profile of microrna-145 in urothelial bladder cancer. Int Braz J Urol. (2013) 39:95–101. doi: 10.1590/S1677-5538.IBJU.2013.01.12 23489501

[19] Li H Huang G Lai Y Ni L Lai Y. A panel of three serum microRNAs as a potential diagnostic biomarker for urothelial carcinoma. Oncol Res Treat. (2022) 45:344–52. doi: 10.1159/000523853 35231919

[20] Wen Z Huang G Lai Y Xiao L Peng X Liu K. Diagnostic panel of serum miR-125b-5p, miR-182-5p, and miR-200c-3p as non-invasive biomarkers for urothelial bladder cancer. Clin Transl Oncol. (2022) 24:909–18. doi: 10.1007/s12094-021-02741-3 35028929

[21] Lin GB Zhang CM Chen XY Wang JW Chen S Tang SY. Identification of circulating miRNAs as novel prognostic biomarkers for bladder cancer. Math Biosci Eng. (2019) 17:834–44. doi: 10.3934/mbe.2020044 31731380

[22] Yuan LQ Zhang T Xu L Han H Liu SH. miR-30c-5p inhibits glioma proliferation and invasion via targeting Bcl2. Transl Cancer Res. (2021) 10:337–48. doi: 10.21037/tcr-19-2957 PMC8798180 35116264

[23] Wu X Chen H Zhang G Wu J Zhu W Gu Y. MiR-212-3p inhibits cell proliferation and promotes apoptosis by targeting nuclear factor IA in bladder cancer. J Biosci. (2019) 44(4):80. doi: 10.1007/s12038-019-9903-5 31502558

[24] Zhang L Zhang Y Wang S Tao L Pang L Fu R. MiR-212-3p suppresses high-grade serous ovarian cancer progression by directly targeting MAP3K3. Am J Transl Res. (2020) 12:875–88. PMC7137041 32269720

[25] Cao HL Liu ZJ Huang PL Yue YL Xi JN. lncRNA-RMRP promotes proliferation, migration and invasion of bladder cancer via miR-206. Eur Rev Med Pharmacol Sci. (2019) 23:1012–21. doi: 10.26355/eurrev_201902_16988 30779067

[26] Huang B Zhai W Hu G Huang C Xie T Zhang J. MicroRNA-206 acts as a tumor suppressor in bladder cancer via targeting YRDC. Am J Transl Res. (2016) 8:4705–15. PMC5126315 27904673

[27] Yuan X Yu L Li J Xie G Rong T Zhang L. ATF3 suppresses metastasis of bladder cancer by regulating gelsolin-mediated remodeling of the actin cytoskeleton. Cancer Res. (2013) 73:3625–37. doi: 10.1158/0008-5472.CAN-12-3879 23536558

[28] Meng S Fan X Zhang J An R Li S. GJA1 expression and its prognostic value in cervical cancer. BioMed Res Int. (2020) 2020:8827920. doi: 10.1155/2020/8827920 33299882 PMC7709497

[29] Yin YW Liu KL Lu BS Li W Niu YL Zhao CM. RBM24 exacerbates bladder cancer progression by forming a Runx1t1/TCF4/miR-625-5p feedback loop. Exp Mol Med. (2021) 53:933–46. doi: 10.1038/s12276-021-00623-w PMC8178337 34021255

[30] Lu Q Liu T Feng H Yang R Zhao X Chen W. Circular RNA circSLC8A1 acts as a sponge of miR-130b/miR-494 in suppressing bladder cancer progression via regulating PTEN. Mol Cancer. (2019) 18:111. doi: 10.1186/s12943-019-1040-0 31228937 PMC6588875

[31] Yan Y Li J Ye M Li Z Li S. Tropomyosin is potential markers for the diagnosis and prognosis of bladder cancer. Dis Markers. (2022) 2022:6936262. doi: 10.1155/2022/6936262 35734544 PMC9208974

